# Supplementary material for: Olive Leaf Processing for Infusion Purposes
Source: Foods. 2023 Jan 30;12(3):591. doi: 10.3390/foods12030591 (PMC9914354; doi:10.3390/foods12030591)
Supplement: Supplementary file 1 [file foods-12-00591-s001.zip › foods-2115710-supplementary.pdf]

Table S1. The coefficient values of the Page model for the drying of olive leaves.

**Drying equipment**	**Temperature (T) ºC**	**Drying co-efficient (*k*) s-1**	**Coefficient (*a*)**	**Coefficient of determination (R^2)**
Oven	40	59.40	0.21	0.99
	50	469.44	0.61	0.99
	60	969.12	0.61	0.99
	70	8584.56	0.51	0.99
Infrared	40	297.00	0.45	0.98
	50	1813.68	0.62	0.99
	60	16340.04	0.75	0.98
	70	71269.20	0.34	0.99
